# An evidence-based, structured, expert approach to selecting essential indicators of primary care quality

**DOI:** 10.1371/journal.pone.0261263

**Published:** 2022-01-18

**Authors:** Sylvia J. Hysong, Kelley Arredondo, Ashley M. Hughes, Houston F. Lester, Frederick L. Oswald, Laura A. Petersen, LeChauncy Woodard, Edward Post, Shelly DePeralta, Daniel R. Murphy, Jason McKnight, Karin Nelson, Paul Haidet

**Affiliations:** 1 Center for Innovations in Quality, Effectiveness, and Safety (IQuESt), Michael E. DeBakey VA Medical Center, Houston, Texas, United States of America; 2 Section of Health Services Research, Department of Medicine, Baylor College of Medicine, Houston, Texas, United States of America; 3 Department of Biomedical and Health Information Sciences, University of Illinois at Chicago, Chicago, Illinois, United States of America; 4 Center of Innovations in Chronic Complex Healthcare, Edward Hines Jr VA Medical Center Hines, Hines, Illinois, United States of America; 5 Department of Psychology, Rice University, Houston, Texas, United States of America; 6 Department of Health Systems and Population Health Science, University of Houston College of Medicine, Houston, Texas, United States of America; 7 VA HSR&D Center for Clinical Management Research, Ann Arbor, Michigan, United States of America; 8 VA Greater Los Angeles Healthcare System, Los Angeles, California, United States of America; 9 Department of Primary Care and Population Health, Texas A&M Health Science Center, Bryan, Texas, United States of America; 10 VHA Primary Care Analytics Team, VA Puget Sound Healthcare System, Seattle, Washington, United States of America; 11 Penn State Health West Campus Health and Wellness Center, Hershey, Pennsylvania, United States of America; University of Technology Sydney, AUSTRALIA

## Abstract

**Background:**

The purpose of this article is to illustrate the application of an evidence-based, structured performance measurement methodology to identify, prioritize, and (when appropriate) generate new measures of health care quality, using primary care as a case example. Primary health care is central to the health care system and health of the American public; thus, ensuring high quality is essential. Due to its complexity, ensuring high-quality primary care requires measurement frameworks that can assess the quality of the infrastructure, workforce configurations, and processes available. This paper describes the use of the Productivity Measurement and Enhancement System (ProMES) to compile a targeted set of such measures, prioritized according to their contribution and value to primary care.

**Methods:**

We adapted ProMES to select and rank existing primary care measures according to value to the primary care clinic. Nine subject matter experts (SMEs) consisting of clinicians, hospital leaders and national policymakers participated in facilitated expert elicitation sessions to identify objectives of performance, corresponding measures, and priority rankings.

**Results:**

The SMEs identified three fundamental objectives: access, patient-health care team partnerships, and technical quality. The SMEs also selected sixteen performance indicators from the 44 pre-vetted, currently existing measures from three different data sources for primary care. One indicator, Team 2-Day Post Discharge Contact Ratio, was selected as an indicator of both team partnerships and technical quality. Indicators were prioritized according to value using the contingency functions developed by the SMEs.

**Conclusion:**

Our article provides an actionable guide to applying ProMES, which can be adapted to the needs of various industries, including measure selection and modification from existing data sources, and proposing new measures. Future work should address both logistical considerations (e.g., data capture, common data/programming language) and lingering measurement challenges, such as operationalizating measures to be meaningful and interpretable across health care settings.

## Introduction

The purpose of our paper is to illustrate the application of an evidence-based, structured methodology for identifying, prioritizing, and (where appropriate) suggesting new measures of health care quality, using primary health care as a case example. Primary health care provides the principal point of entry for patients into the health care system in America and many other countries, and is often the gateway for initially accessing most other specialties. Research has consistently demonstrated that people in countries and health systems with high-quality primary care exhibit better health outcomes and greater health equity [1]. Without access to consistent primary care to, among other functions, address patients’ preventive care needs and coordinate their health needs across medical subspecialties, (among other functions) minor, preventable health problems often devolve into significant chronic conditions, increasing emergency department utilization, and exponentially multiplying health care costs.

Because of its central role in the workings of the health care system and health of the public, a high-performing, high-quality primary care system is essential; private and public entities alike have devoted considerable resources toward that goal, making it an ideal setting for demonstrating the application of this methodology. For our purposes, we conceptualize primary care as defined by the latest report from the (United States) National Academies of Science Engineering Medicine (NASEM) on implementing high-quality primary care:

*the provision of whole-person*, *integrated*, *accessible*, *and equitable health care by interprofessional teams that are accountable for addressing the majority of an individual’s health and wellness needs across settings and through sustained relationships with patients*, *families*, *and communities*.

*1*

This latest definition is consistent with and builds upon the work of other public and private organizations, such as the United Kingdom’s National Health Service (NHS) [2], the World Health Organization [3], and the Commonwealth Fund. For example, the Commonwealth Fund’s Safety Net Medical Home Initiative identified eight essential characteristics that primary care practices should change to successfully become patient centered medical homes: (1) engaged leadership, (2) quality improvement strategy, (3) empanelment, (4) continuous and team-based healing relationships, (5) organized evidence-based care, (6) patient-centered interactions, (7) enhanced access, and (8) care coordination [4]. Similarly, The Center of Excellence in Primary Care at the University of California San Francisco developed ten building blocks of high-performing primary care, which include the 8 elements identified by The Commonwealth Fund’s Safety Net Medical Home Initiative [5]. The expansion of primary care to include elements such as empanelment and data-driven improvement [4,5], is highlighted in a recent AHRQ white paper on redefining primary care, which shows increasing consensus on the components of high-quality primary care [6]. These and other works (e.g., [7] have served as the foundation for the current NASEM’s vision of primary care, which better incorporates the team-based nature of modern primary health care delivery, and its central role in the community.

As reflected by its modern conceptualization, primary care delivery has evolved over the last few decades, facilitated by significant advances in medicine, technology, and policy. Consequently, measurement frameworks are needed to assess the quality of the infrastructure, workforce configurations, and processes available in primary care practices due to the complexity of primary care. Health-care systems in several countries have embarked on creating such frameworks [8] for primary and specialty care. The NHS’ Quality and Outcomes framework is one such example, organizing their all-encompassing indicator set along clinical, organizational, and patient experience indicator domains [9]. Other countries, such as Australia and Germany have built parallel frameworks, relying on the clinical evidence base for selection [8,10]. In the United States, a plethora of measures now exists to help monitor primary care activities, including the number of appointments, patient load, and clinical performance assessments of patient-related data (e.g., the Healthcare Effectiveness Data and Information Set [HEDIS]). Furthermore, new team-based primary health care delivery configurations such as the Patient-Centered Medical Home (PCMH) [11] and the Veterans Health Administration’s (VHA) Patient Aligned Care Teams (PACT) [12] have accelerated the development of such measures to assess the quality of primary care. In the examples listed above, clinical evidence base and data collection feasibility drive the selection of a given performance measure. This practice contradicts measurement best practices in quality improvement, which prioritize matters of strategic value [13]. Further, improvement on such measures may prove impractical or face limitation of a ceiling effect (e.g., patient portal uptake in underserved patient populations with limited smartphone and internet access). Unfortunately, the byproduct of these practices results in a confusing excess of clinical performance measures.

Such proliferation of performance measures, of both high and low quality, bears unintended consequences on the primary care workforce; for example, in 2014, Kansagara and colleagues [14] reported that primary care staff perceived that some performance measures: “1) led to delivery changes that were not always aligned with PACT principles, 2) did not accurately reflect patient priorities, 3) represented an opportunity cost, 4) were imposed with little communication or transparency, and 5) were not well adapted to team-based care.” Powell and colleagues [15] further suggested that implementing additional performance measures generates non-value-added clinical burden, such as redundant documentation and a tendency to focus on actions that quickly improve scores, as opposed to actually improving patient outcomes. These actions, in turn, often result in inappropriate patient care and decreased patient-centeredness (e.g., lack of focus on patient concerns). Related work illustrates the vast range of unintended consequences for collecting new care quality and performance measures [16,17], ranging from redundant or unnecessary work to clinician dissatisfaction and burnout, leading to an exodus from primary care and worsening primary care provider shortages.

Despite advances in primary care delivery in the last several decades (e.g., team-based care delivery and greater use of electronic health records and patient portals), the fundamental objectives of primary care (e.g., improving population health) and the nature of the work typically performed in primary care (e.g., patient assessment and treatment of common and chronic conditions, medication management, preventive care, coordination of care for complex patients) have remained largely unchanged [18–20]). Given the host of unintended consequences associated with large performance measure frameworks, this raises questions regarding the need for the recent proliferation of novel quality and clinical performance metrics. Further, with the exponential growth of readily available digital health information in the last decade, distinguishing meaningful, appropriate, and high-quality performance measures from irrelevant, redundant, or unrepresentative performance data quickly becomes a necessary yet time-demanding undertaking. Such concerns suggest that the extent to which primary care clinics are delivering on the fundamental promise of primary care could be assessed more effectively through a tailored set of quantifiable performance measures prioritized according to value. A systematic approach is thus needed to identify, select, and prioritize said measures.

### Evidence-based approaches to curating primary care measure sets

Ideally, scientific evidence informs the development, validation, and ultimately the application(s) of performance measures. Specifically, good performance measures propose criteria by which the performer is to be evaluated. A set of performance measures, however, can serve multiple purposes, including assessment of similar criteria across different settings (e.g., multiple organizational types) and different people (e.g., multiple subjects). Further, performance measures are adaptable in that they can serve more than one purpose or interpretation of their criteria. For example, blood pressure and heart rate measures (e.g., heart rate variability, beat-to-beat intervals) can indicate an individuals’ cardiovascular health [21]. However, this same set of measures in a healthy sample of adults can also be used to infer an individuals’ level of stress and experienced workload [22,23]. Rather than flood the literature with additional measures–which are resource-intensive to develop, validate, and integrate into clinical practice–we detail the adaptation of an evidence-based approach originally created for performance-measure development, and demonstrate its application to select (modify, when appropriate) and rank existing primary care measures according to their value in a given context, which in the current case study is primary care clinics.

The Productivity Measurement and Enhancement System (ProMES) is a structured, evidence-based, comprehensive productivity improvement approach; originating from the industrial/organizational psychology and management literatures, it is firmly grounded in motivational theory, the science of collective sensemaking, and performance measurement [24–30]. ProMES has been used in multiple countries such as Germany, Netherlands, Hungary, Sweden, Australia, and the United States, and has been used successfully in multiple industries including police work, university research, manufacturing, and healthcare settings, including primary care [24,25,31–33]. Through a facilitated focus-group based process, these measures are defined, weighted, and prioritized to create indicators of both overall effectiveness and specific aspects of daily work. This alignment helps individuals and teams to focus their effort more clearly on the most important aspects of their work (i.e., clinical performance) resulting in greater productivity (i.e., how effectively an organization uses its resources to achieve its goals), reduced stress, and less waste of effort [34].

Previous research in intensive care unit and mental health settings have shown successful use of ProMES to improve efficiency and quality of care. For example, in studies conducted with a German mental health hospital, the ProMES methodology was used across organizational levels: the top management team, samples of nurses, and three samples of technicians. As a whole, results indicated that overall productivity scores for participating units were .78 standard deviations higher, on average, after implementation [35]. More recently, ProMES was used in primary care teams within the VA to improve coordination; in this study, teams configured according to PACT recommendations, and who more faithfully followed the ProMES process exhibited improvements in key coordination processes (e.g., appointments starting on time, clinical reminder completion) [24]. A separate implementation evaluation of ProMES at different VA primary care facilities showed significant improvements in overall coordination compared to baseline and to administratively matched controls [36]. Broader meta-analytic work examining the effectiveness of ProMES on improving productivity found a large overall effect size (δ = 1.44); with even higher effect sizes within particular subgroups (as high as δ = 2.21).

One unique feature of ProMES is that the resulting measures include non-linear functions that explicitly tie performance levels on a given measure to its contribution to the organization’s values; in this way, the application of ProMES yields a more nuanced approach to prioritizing measures than simple linear weights, while allowing direct comparison(s) between measure(s) [37]. In short, ProMES’ firm theoretical grounding, participatory development process, non-linear prioritization functions, single index of productivity, and decades of research demonstrating its effectiveness position it as a highly viable method for selecting and curating a targeted primary care performance set.

### Study objective

Our objective in this paper is to illustrate the application of ProMES to select and curate a targeted set of primary care quality measures, prioritized according to their overall contribution and value to primary care. Although the illustrative case below is part of a larger project [34] where primary care measures were selected from existing care metrics and data sources available from the United States Department of Veterans Affairs (VA), the measures are not specific to VA and can apply to other primary care settings. Herein we describe the process by which we applied the ProMES methodology to select primary care performance measures and present the final set of measures resulting from the process.

## Methods

### Ethics statement

This study was reviewed and approved by the Baylor College of Medicine Institutional Review Board (IRB), protocol #H-42358. Human subjects consent was waived by the IRB, as no human subjects were involved: the participants described below were deemed subject-matter experts, not subjects of the study.

### Participants

The first step in the ProMES approach was the selection of our subject matter expert (SME) panel, known in ProMES parlance as a design team. We invited SMEs who met the following criteria: (1) filled gaps in the project team’s knowledge base, (2) possessed significant experience or a leadership role in primary care settings, and (3) were highly knowledgeable of and experienced with performance measures and/or electronic health record (EHR)-sourced data. To ensure representativeness in the design team, we additionally ensured the team consisted of experts from both inside and outside the VHA (our principal source of measure data) and represented multiple stakeholder groups including active primary care clinicians, administrators, performance measure data managers, and measurement experts. Our final team consisted of nine subject matter experts in addition to our project team. S1 File lists the members of the expert panel and details their qualifications.

### Procedure

We adapted the ProMES steps involved in developing a performance measurement system to accomplish our objectives. Traditional ProMES methodology involves forming a design team of SMEs, developing objectives, generating performance indicators, and generating assessments of value for each indicator (a process called contingency development). Each step is described below. For this project, we adapted the indicator development process (Step 2) so that our design team selected from existing measures, rather than generating new ones. We identified potential indicators from an existing pool of primary care measures available on a national level through the VHA. Each potential indicator/existing measure was thoroughly vetted for quality against ProMES indicator quality criteria in order to mirror the methodologically rigorous characteristics of performance indicators *generated* through ProMES.

#### Step 1: Form design team

The design team consisted of nine subject matter experts in addition to our study team, selected as described in the Participants section, above.

#### Step 2: Identify clinical performance objectives

A fundamental assumption of ProMES is that effective performance measurement cannot be accomplished without knowing what overarching performance objective is sought. Our design team was led by project team facilitators in a 1-day workshop to identify the clinical objectives for primary care. According to ProMES, objectives are defined as the essential things a unit (i.e., a primary care team) does to add value to the organization; in other words, objectives are the main result (and associated characteristics) of the primary care team’s work (e.g., amount, quality, timeliness). Examples of health care objectives include patient care performed according to quality standards, effective patient throughput, and personnel allocation matched to patient workload. The key question the design team was charged with answering was “what is the primary care team trying to accomplish when it delivers care?” The facilitators guided the design team to arrive at three to six objectives that met the following criteria: (1) was stated in clear terms, (2) designed so that if exactly that objective was accomplished, the facility (and thus the patient) would benefit; (3) the set of objectives cover all important aspects of clinical care; (4) was consistent with broader objectives (such as AHRQ conceptualizations of primary care); and (5) leadership was committed to each objective. Objectives were compared against these criteria to ensure their utility in facilitating the subsequent steps of the process.

#### Step 3: Select performance indicators

Having identified primary care teams’ healthcare delivery objectives, the next step was to identify indicators for these objectives. In the classic ProMES process, these indicators would be generated *de novo*, rather than selected from a set. However, as such an abundance of measures already exist for primary care, the ProMES process was used slightly differently, yet innovatively–to cull, rather than generate, indicators of performance. For each objective, the design team answered the following question: “How would you show that the primary care team is meeting the stated objective?” To accomplish this, the design team received a document containing names, sources, and operational definitions of all outpatient clinical performance measures currently tracked by the VHA that were available at the team level. To ensure generalizability beyond the VHA health care system, any measures that captured VHA-specific processes or policies (e.g., homeless veteran measures; veteran patient portal usage) were excluded. Measures for the design team’s consideration were drawn from VHA’s principal outpatient clinical data sources and reports: VHA’s Corporate Data Warehouse, the principal repository of clinical and administrative data; the Strategic Analytics for Improvement and Learning (SAIL) system, VHA’s system for summarizing hospital performance, which includes the HEDIS measures used by most medical practices nationwide; and the Electronic Quality Measures (eQM), which provides, real-time, 100% sampling of patients for measure calculation.

The design team selected indicators via a two-step process. Design team members received documents describing the candidate measures two weeks in advance of the aforementioned day-long workshop so they would have adequate time for review. As part of the day-long workshop, the facilitators led the design team through a preliminary group voting process to identify the strongest candidate measures for each objective. In the weeks following the workshop, the team met virtually in a series of weekly 1 to 1½-hour facilitator-led meetings for up to a total of approximately 12 hours of meeting time. For each objective, the design team reviewed each candidate measure that survived the preliminary voting process. The team collectively narrowed down the list to a targeted set of 4–6 performance indicators per objective that captured the extent to which the primary care objectives were being achieved. Each indicator needed to meet multiple criteria regarding its validity, comprehensiveness, impact, feasibility, and usability.

#### Step 4: Prioritize indicators by developing contingencies

Indicators provide information about what is valued in performance (e.g., the number of days between a fecal occult blood test (FOBT) order and a scheduled colonoscopy signals timeliness in care delivery); however, what level of performance is acceptable, or how much a given level of improvement is valued (Is an average of 7 days acceptable? How much worse is 8 days? 10? How much better is 5 days?) is provided by a ProMES tool called contingencies. One of the most novel aspects of ProMES is the use of contingencies. Contingency development generates a function for each indicator that shows how much the different amounts of the indicator (e.g., 5 vs. 7 days) contribute to overall effectiveness. By relating each indicator to overall effectiveness, they are put on the same measurement scale, which ranges from -100 to +100. Thus, the various indicators can be directly compared, prioritized, and combined into a single measure if needed. Most importantly, it reflects an explicit statement of what elements of primary care are valued, and what level of primary care-related performance is expected and valued by the team and the facility.

**Fig 1:**
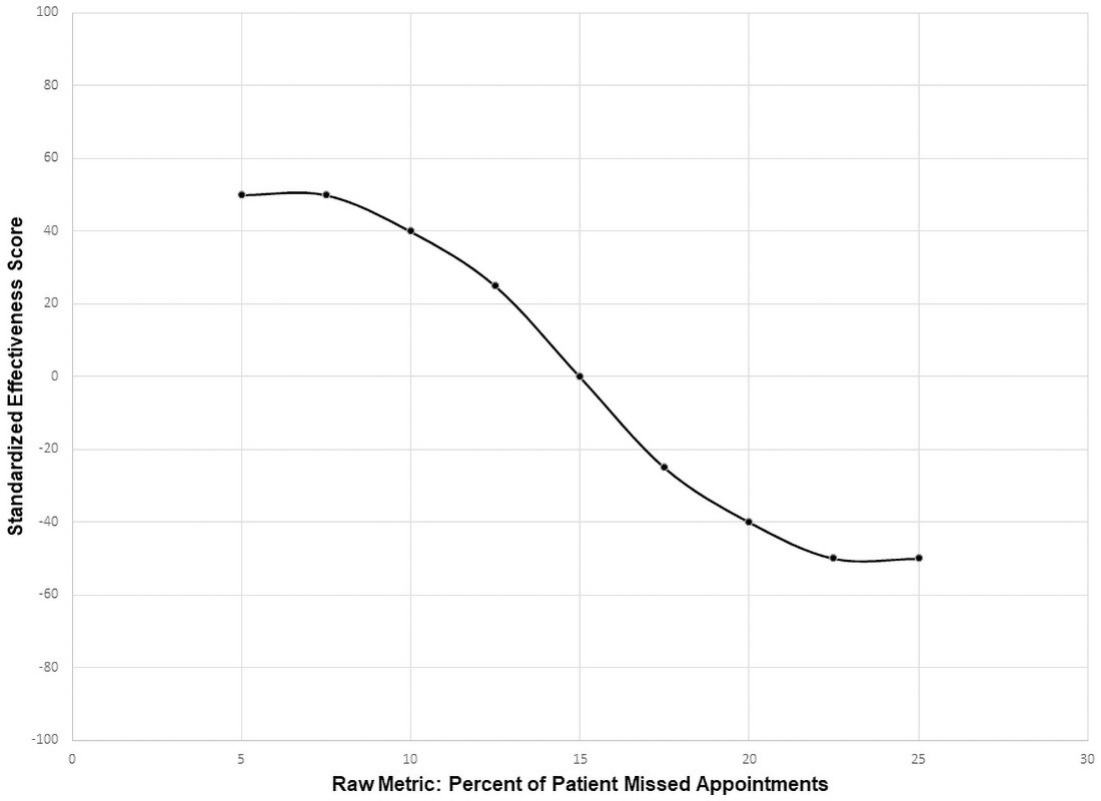
Example contingency curve.

Once the design team selected the indicators for each of the objectives, the classic ProMES approach was followed (see Pritchard et al., 2011 [28] for details). In the classic ProMES approach, the design team determines three critical scores for each of the indicators: realistic minimum, realistic maximum, and the minimum level of acceptable performance. Fig 1 shows an example graph for an example indicator: percent of patients who missed appointments during the reporting period. In this example, the design team for the clinic would decide the realistic minimum (5% in this example) and the realistic maximum (25% in this example). Next, the minimum level of acceptable performance is determined. In this example, the minimum level of acceptable performance is when 15% of patients miss their appointments during the reporting period.

Once these three critical scores are identified, they are mapped by the design team to a standard effectiveness scale, with a maximum possible score of 100, and a minimum possible score of -100. The minimum level of acceptable performance for any indicator always corresponds to a score of zero on this effectiveness scale (see Fig 1). The realistic maximum score of the indicator deemed as yielding the greatest gains to the organization if perfect performance could be achieved always corresponds to a score of 100 on the effectiveness scale. Realistic maxima and minima of the remaining indicators are then mapped onto the effectiveness scale relative to this maximum of 100.

In our study, the design team convened for a total of 6–8 hours (in multiple 1 to 1½-hour virtual meetings) to develop a contingency function for each indicator. In doing so, members of the design team identified the maximum and minimum possible levels of performance, the minimum expected performance level, and scaled the various levels of performance to a common measure of effectiveness for each indicator. This was accomplished with the aid of the principal investigator serving as facilitator who, for each indicator, elicited discussion about each of the aforementioned data points, and guided each discussion to consensus through real-time, iterative polling of the design team.

## Results

### Objectives

The design team identified three fundamental objectives for delivery of high-quality primary care:

- Objective 1. Ensure patient has appropriate access to preventive, acute, or chronic health care services when needed.
- Objective 2. Build a trusting, effective, sustained partnership between the health-care team, the patient, and his/her caregiver(s) towards shared goals.
- Objective 3. Deliver safe and effective care that comprehensively addresses a given patient’s particular ecological, biological, and/or psychosocial needs.

A fourth objective, proactively monitor and engage with patient population(s) to optimize their treatment plans and health behaviors towards improved overall health, was originally identified. However, as the design team selected performance indicators for each of the objectives, it became evident that the fourth objective was subsumed under the other three objectives. Thus, the design team opted for a more succinct set of objectives and instead allowed for a slightly greater number of indicators in total, to capture this aspect of the primary care performance domain.

### Indicators and contingency curves

The design team selected sixteen performance indicators from the 44 pre-vetted measures that already exist in three different data sources for primary care, as outlined in Step 3. One indicator, Team 2-Day Post-Discharge Contact Ratio, was selected as an indicator for both Objective 2 and 3. In addition, contingency curves were created for each of the indicators using the contingency functions developed by the design team as described in Step 4. Table 1 lists the primary care performance indicators selected by the design team, organized by objective. In Table 1, Team 2-Day Post-Discharge Contact Ratio appears under both Objective 2 and 3.

**Table 1:** Consolidated, summary table of indicators including suggested, by objective.

Indicator	Definition
*Objective 1*: *Access*	
1. Average 3rd Next Available Appointment in PC Clinics	The average waiting time in days between a completed appointment and the 3^rd^ Next Available Appointment slot for each primary care clinic. A snapshot is taken on the first day of each month for the prior month’s activity. The wait times in days until the 3^rd^ Next Available Appointment is averaged for completed appointments.
2. Established Primary Care Patient Average Wait Time in Days	The average number of calendar days between an established patient’s PC completed appointment and earliest of three possible preferred (desired) dates from the completed appointment date.
3. Urgent Care Utilization Rate	This measure is based on a question which asks patients to rate their primary care provider, after a qualifying visit on a 0 to 10 scale with 0 being the worst possible and 10 being the best. This measure is the percentage of patients’ responses of a 9 or 10 (the top two categories).
4. New Primary Care Patient Average Wait Time in Days	The average number of calendar days between a new patient’s PC completed appointment and the earliest of three possible preferred (desired) dates from the completed appointment date.
5. Total Inbound PC Secure Messages to Total Outbound PC Secure Messages (Ratio)	This measure is a ratio representing the total number of secure messages sent by a patient assigned to a given primary care team divided by the total number of secure messages sent from a primary care team member to a patient assigned to that primary care team during the reporting period.
6. Average Consult for Community Care*	Measure showing average number of referrals and appointments within and outside a specified hospital system to gain access to appropriate specialty service(s).
7. Timeliness of Community Care Referrals*	Timeliness of referrals to specialty care service(s) where an appropriate number of days is assigned to each specialty.
8. Comprehensive Preventative Visits*	Completed preventative care appointments by a patient assigned to a given team during the reporting period. Preventative services could include, but are not limited to, vaccinations, cancer screenings, mammograms, colonoscopies, or stool testing.
9. Urgent Care Utilization Rate (Adjusted for clinical reason)*	Measure should capture why patients utilize urgent care. Utilization rate could be high because patient received ineffective care, do not have access to PCP, or because necessary and reflects good coordination.
*Objective 2*: *Clinician-Patient Partnership*	
1. Team 2 Day Post Discharge Contact Ratio**	This measure represents the percentage of patients assigned to a given primary care team who were contacted within two days of being discharged (DC) from inpatient care. The post discharge contact is only counted if the individual contacting the patient has a team role of administrative associate, care manager, clinical associate, designated women’s health primary care provider, clinical pharmacist, physician-attending, or primary care provider. Patients are excluded from this measure if they are discharged from an observation specialty and/or are readmitted within two business days to any healthcare facility.
2. Patient’s Satisfaction Rating of Primary Care Provider	This measure is based on a question which asks patients to rate their primary care provider, after a qualifying visit on a 0 to 10 scale with 0 being the worst possible and 10 being the best. This measure is the percentage of patients’ responses of a 9 or 10 (the top two categories).
3. Patient-Centered Medical Home Stress Discussed	This measure comes from a question which asks the patient if, “in the last 6 months, did anyone in this provider’s office talk to you about things in your life that worry you or cause you stress”? The measure reflects the percentage of patients who responded “yes” to the question.
4. Average Effective Partnership Rating*	Average rating of providers’ effective partnership. Captured by developing an “Effective Partnership Rating Scale.”
5. Average Team Trust Rating*	Average rating of team trust. Patients could rate how much they trust each member of their assigned primary care team, as well as the overall team. In addition, primary care team members would also rate how much they trust each of their team members, as well as the overall team. This measure is similar to Consumer Assessment of Healthcare Providers and Systems (CAHPS) Q11, Q14, and Q15 on care coordination and person-centered care but CAHPS does not capture trust.
6. Effective PC Team Ratio*	This measure captures whether a patient’s primary care needs were met by someone from the patients assigned team, when needed. The measure is calculated with the following formula: Number of primary care team encounters WOT (while on team) with patients assigned team member divided by number of primary care team encounter WOT plus the number of ER/Urgent care encounters excluding ED visits in the denominator. This item is similar to PACT 19 in PACT Compass, except PACT 19 includes ED visits.
7. Continuity Care Ratio*	Year over year retention rate with patient panel. Compare across provider, where higher rate means patients are choosing to stay with the provider.
*Objective 3*: *Technical Quality*	
1. Hospital-wide all cause 30-day Readmission Rate^†^	Rate of unplanned readmissions in the 30 days after discharge from a hospitalization. Rate is derived from a composite of five statistical models, built from groups of hospitalizations that are clinically related: Cardiorespiratory, Cardiovascular, Medicine, Neurology, and Surgery/Gyn. The measure does not count planned readmission. This measure is designed to provide aggregate and detailed views of the data to assist managers and clinicians in identifying potential gaps when transitioning patients through different stages of the recovery processes.
2. Ambulatory Care Sensitive Conditions (ACSC) Hospitalizations Rate Per 1000 Patients	Hospitalizations due to ACSCs such as hypertension, congestive heart failure, and pneumonia can typically be avoidable or preventable if ambulatory care is provided in a timely and effective manner. It has been well established that effective primary care is associated with lower hospitalization due to ACSCs. This rate is calculated by AHRQ using state population and the equation is ACSC hospitalizations divided by ACSC population. A similar option to calculate the ACSC hospitalization rate per 1,000 patients is by calculating the number of inpatients with a principal diagnosis of ACSC divided by the number of total patients with any diagnosis of ACSC.
3. Diabetes Electronic Composite Measure^†^	This measure is a composite of the “Diabetes Patients with HbA1c Poor Control” measure and the HEDIS measure “Diabetes Mellitus—Outpatient: HbA1c Annual Testing” which is the number of patients between 18 and 75 years of age who have had HbA1c testing within the measurement year.
4. Diabetes Patients with HbA1c Poor Control	This measure represents the number of patients diagnosed with diabetes mellitus between the ages of 18 and 75 whose HbA1c score is greater than 9 or who show no evidence of having their HbA1c tested within the last year.
5. Team 2 Day Post Discharge Contact Ratio**	This measure represents the percentage of patients assigned to a given primary care team who were contacted within two days of being discharged (DC) from inpatient care. The post discharge contact is only counted if the individual contacting the patient has a team role of administrative associate, care manager, clinical associate, designated women’s health primary care provider, clinical pharmacist, physician-attending, or primary care provider. Patients are excluded from this measure if they are discharged from an observation specialty and/or are readmitted within two business days to any healthcare facility.
6. Controlling High Blood Pressure^†^	This is the number of patients between the ages of 18 and 85 with a diagnosis of hypertension within the first six months of the measurement year who are later found to have: + A blood pressure of less than 140/90 for outpatient patients aged 18–59; + A blood pressure of less than 140/90 for outpatients aged 60–85 with a diagnosis of diabetes mellitus (DM); Or +A blood pressure of less than 150/90 for outpatient patients aged 60–85 without a DM diagnosis.
7. Statin Medication for Patients with Cardiovascular Disease^†^	This measure is the number of male patients age 21–75 and female patients age 40–75 with cardiovascular disease who had at least one dispensing event for a high or moderate-intensity statin medication (as defined by HEDIS) during the measurement year.
8. Effective Continuation Phase Treatment for depression	This percent is the number of patients over age 18 with a diagnosis of depression who received greater than or equal to 180 days of antidepressant medication through 231 days after the index prescription start date, divided by the number of patients with a diagnosis of depression newly treated with antidepressant medication.
9. Renal Testing for Nephropathy	This measure consists of the percentage of diabetes patients between the ages of 18 and 75 who had a nephropathy screening test during the measurement year.
10. Consult for Community Care*	Percent of referrals to community care that were successfully completed (numerator: number of referrals to community care for which a response from the community care provider was logged into the referring provider’s EHR; denominator: number of referrals to community care logged in the referring provider’s EHR).
11. Timely Clinic Communication*	Mean clinic response time in days to *patient* requests for clinical information and/or the mean clinic response time in days to *provider* requests for clinical information.
12. Missed Opportunities for Care Coordination*	Percent of charts where missed opportunities for care coordination were identified in random peer review process. Could also be measured with number of true trigger positives, e.g., Positive FOBT–no follow up action (colonoscopy) within 60 days, Mammogram with BIRADS 0,4,5 –no follow up action (ultrasound, repeat mammogram, breast biopsy, breast MRI, breast surgery, oncology visit) within 60 days.
13. Average PCP Safe and Effective Care Rating*	This measure captures patients’ average perception of the safe and effective care provided by their primary care provider. Patients rate their primary care provider on a “Safe and Effective Care Scale” which captures patients’ perceptions of whether Objective 3 is being met.
14. Decrease Inappropriate Antibiotic Prescribing*	Number of patients where antibiotics were prescribed for viral URI symptoms divided by number of patients with viral URI symptoms.

Notes:

* Denotes indicators that did not exist at the time of the SME focus groups, but were nonetheless suggested by the design team as important aspects to assess.

** Team 2-Day Post-discharge contact ratio was identified by the subject matter experts as a key indicator for both Objectives 2 and 3. PC = primary care. PCP = primary care provider.

^†^Denotes metrics adopted or adapted from systems external to VHA (e.g., National Quality Forum, Healthcare Effectiveness Data and Information Set[HEDIS]).

### Gaps in the primary care performance measurement domain

The ProMES process is meant to guide measurement development or selection that yields a concise yet comprehensive list of indicators that contribute to an organization’s overall effectiveness [29,35]. The design team identified areas of primary care performance that were not fully captured by the 16 existing indicators; therefore, the design team suggested additional indicators that are not currently measured but would bridge the criterion gaps. This gap was especially evident in Objective 2, where the design team was only able to select three relevant indicators from the existing measures. Table 1 lists these suggested indicators in brief.

## Discussion

Using primary healthcare as an illustrative example, thist paper details the application of an evidence-based approach to select a curated set of healthcare quality indicators, prioritized according to their overall contribution and value to healthcare organizations. In our primary care example, our, o design team of nationally recognized SMEs collectively identified three primary care delivery objectives, selected 16 currently existing indicators of primary care quality directly tied to these objectives, and called for 13 additional indicators requiring further development to address current gaps in the primary care performance measurement domain. The indicators were selected independent of clinic size or configuration, so that clinics of many configurations (e.g., public vs. private, large vs. small, rural vs urban, team-based vs. traditional) could benefit from their use.

Results speak to the type of indicators readily available for each stated objective of care. Our first care objective, centered around access to care, highlights the overall importance of primary care facilities to maintain sufficient capacity. In robustly assessing this care objective, indicator(s) for this objective also capture(s) insufficient or ineffective access to primary care by capturing patients’ visit(s) to the emergency department instead of their primary care clinic for their care. Interestingly, our second care objective, covering the importance of maintaining a trusting partnership with the patient, contained relatively few existing indicators, primarily due to the challenges inherent in self-report data and lack of available objective measures for processes of care delivery face to face (e.g., building rapport). Finally, our third objective of achieving technical quality contains an abundance of measures, reflecting the clinical, condition-specific origins of the performance measurement movement in health care. Measures for these objectives revolve around evidence-based processes for the treatment of highly prevalent chronic physical and mental conditions most commonly managed in the primary care setting; similar to the access objective, an additional facet captures ineffective technical quality through hospitalizations resulting from ambulatory care sensitive conditions and hospital admissions. In turn, our study revealed the existing discrepancies between ideal and current measurement of care quality. Specifically, although assessing the quality of less tangible but essential activities such as disease education and shared decision-making in the context of relationship building with patients would ideally form a significant proportion of quality care measurement, a disproportionate number of existing measures focused on the more readily measured technical competencies in clinical practice (Objective 3). A balanced number of indicators across all three objectives would comprise a more comprehensive set of care quality measures.

### Implications

Our illustrated adaptation of ProMES to quality improvement in healthcare carries both scientific and practical implications. Scientifically, our paper adapts a structured approach from outside health care to provide a structured means of selecting and prioritizing healthcare quality indicators, thus expanding the existing health-care literature addressing quality indicator frameworks and development methods. For example, prior research has advanced the application of several frameworks to categorize clinical indicators, including clinical categories [38], proposed core dimensions of primary care, and Donabedian’s classic structure/process/outcome framework [39–41]. Methods of measure development and composite aggregation (such as clinical expert consensus, cost, and clinical complexity weights) have also been proposed [42]. However, once a measure is defined, operationalized, and a process for data collection identified, we are not aware of a systematic approach for deciding which measures to include in a curated set, or to assess how much importance to assign to each measure in the set. Further, various efforts exist to assess technical proficiency and patient-related outcomes; [39] however, without guidance for selecting, implementing, and interpreting their proposed measures, their subsequent application as proxies for primary care quality and team effectiveness may be misguided. Guidance in terms of measure selection, implementation, and interpretation are needed to further guide assessing primary care effectiveness.

Practically, performance measures selected and prioritized by their value in primary care using our modifications to ProMES help clarify where to best focus efforts, making implementation of targeted care quality improvement initiatives more effective. For example, by deriving high-value metrics, organized by objective with quantifiable prioritization, we anticipate the set of indicators identified in our case study could apply to a diverse set of stakeholders, including but not limited to policy-makers, primary care clinicians, and administrators in healthcare organizations. Furthermore, as more primary care settings transition toward a team-based delivery approach, we believe our curated set of indicators provide more meaningful, appropriate, and high-quality assessments of primary care team effectiveness, enabling low-resource settings (such as Federally Qualified Health Clinics [FQHCs]) to improve the care they deliver [43].

In addition to the viability of the process demonstrated herein, the measures identified by the SMEs bear implications to the scientific and primary care clinical and scientific communities. Practically, our proposed measure set establishes precedent for communicating policy explicitly within care quality measurements. Namely, in regard to care quality among primary care clinics, our targeted manner of measurement explicitly prioritizes the value of each care measure in their overall contribution and value to primary care. In practice, results of using these measures could translate to greater actionability for quality improvement initiatives in the primary care setting. Further, the proposed measures could be adopted to evaluate clinics who wish to pursue certain types of certification, such as PCMH certification through the Joint Commission. Based on currently available data collection techniques, the set of measures presented in our findings offers a less obtrusive, and thus less resource-intensive manner by which team-based primary care quality can be ascertained in that it relies on existing sources of quality data and methods of data capture.

Theoretically, our measures and their respective objectives provide clear domains for measurement of primary care quality that are consistent with modern conceptualizations of primary health care, and which could lead to quality improvement interventions that are better aligned with the goals and the promise of primary care.

### Limitations

Our resulting set of measures is necessarily limited by what was available in existing VHA data sources; indeed, such limits were observed in the need to propose new measures (not presented here) that did not exist in the current data source in order to comprehensively cover the primary care performance domain. Thus, in the current measure set, outcome measures are overrepresented relative to process measures, whereas the reverse is true in the proposed measures.

Although leveraging available measures avoids several unintended consequences involved in care quality measure creation, existing data sources have several documented problems. First, use of electronic health record data for research purposes is often questioned due to completeness and quality of data entered. Second, sampling strategies used to collect more subjective measures of care quality (e.g., patient satisfaction) collect data from a sub-set of a clinical teams’ patient panel and may not fully represent the team’s provision of care. Although no data source is perfect, we hope to target efforts on improving the quality of health systems by building upon existing techniques rather than inventing new methods of data capture. Furthermore, not all practices or systems have the infrastructure needed to measure, capture, and utilize the data to measure care quality. Some practices or systems may not have the resources or have competing resource demands, such as needing to invest resources in equipment or buildings rather than performance measurement. Funding initiatives for practices that want to adopt a PCMH model should provide sufficient incentives to significantly reform infrastructure including a measurement system to measure care quality.

A core assumption of this paper is that the primary care performance objectives result in patients’ improved health and that they align with patients’ goals. Moreover, we are mindful of patient centered issues and needs that are not captured by the objectives, such as the healing comfort a healthcare professional can provide. Future efforts should more explicitly capture the needs of the patient when identifying clinical performance objectives and indicators, as exemplified in several of the measures suggested by the SMEs.

Finally, psychometric procedures have not been conducted on the selected measures as a set to confirm statistically that the selected indicators indeed load onto their respective objectives. Obviously, this check cannot be performed on the proposed measures that do not, as of yet, exist.

### Conclusions and future directions

We conclude that through the use of an evidence-based approach to performance measure development, a targeted set of performance measures can be curated to track progress toward specific objectives in delivering high-quality primary care. The measures identified by the SMEs provide an actionable catalogue that can serve as a first step toward interoperability of electronic health record systems. Future work toward this goal should address both logistical considerations (e.g., data capture, common data/programming language) and lingering measurement challenges, such as the best way to operationalize these measures for teams working in complex and shifting situations (e.g., rotating team members).

## Supporting information

S1 File Subject matter expert credentials and biographies. (DOCX)
