# Supplementary material for: An evidence-based, structured, expert approach to selecting essential indicators of primary care quality
Source: PLoS One. 2022 Jan 18;17(1):e0261263. doi: 10.1371/journal.pone.0261263 (PMC8765671; doi:10.1371/journal.pone.0261263)
Supplement: S1 File — (DOCX) [file pone.0261263.s001.docx]

Design and Research Team Members

# Design Team Roster

### Shelly De Peralta, DNP

Dr. Shelly De Peralta is a full-time nurse practitioner with doctoral-level training in nursing practice. She has served as a registered nurse for nearly 24 years. Her area of interests includes improving the quality of care provided by nurse practitioners. With the introduction of ongoing professional practice evaluation (OPPE) and Focused Professional Practice Evaluation (FPPE), she assumed a leadership role in overseeing the Professional Practice Evaluation (PPE) program at VA Greater Los Angeles (GLA). The program’s focus is towards capturing and measuring the delivered quality of care by Advanced Practice Registered Nurses (APRN). With the introduction of Strategic Analytics for Improvement and Learning (SAIL) metrics, both outpatient and inpatient performance improvement activities reports are delegated to Dr. De Peralta. Shelly reports progress to the Chief of Staff and Nurse Executive offices. She is responsible for understanding measure specifications, evaluating current processes, bringing stakeholders on board and refining/ adjusting/redesigning the processes necessary to improve quality of care. She also participates in the GLA SAIL steering committee reporting to the Deputy Chief of Staff.

### Adol Esquivel, MD, Ph.D.

Dr. Adol Esquivel joined CHI St. Luke’s Health System as the Director of Clinical Effectiveness and Performance Measurement in 2010, overseeing the management, integration and coordination of St. Luke's clinical performance measurement systems. Dr. Esquivel was promoted in 2012 to Assistant Vice President of Performance Measurement overseeing the Houston facilities, and in 2017, he was named Division Director for Clinical Analytics overseeing the Texas facilities. He is currently responsible for data quality and statistical analysis of priority populations, clinical abstraction services and clinical databases for quality reporting. Dr. Esquivel ensures that accurate analytic methodologies are used to develop and transmit internal and external clinical quality performance measures, as well as the creation of hospital and safety trending systems.

### Joe Francis, MD, MPH

Dr. Joe Francis has served as the Chief Improvement and Analytics Officer for the Veterans Health Administration since July 2019. In this role, he serves as a principal advisor to the Executive in Charge, Office of the Under Secretary for Health and the Principal Deputy Under Secretary for Health. He leads a multi-disciplinary team responsible for using VA’s comprehensive electronic health record to track health system performance and provide clinicians and managers with analytic and information tools that support improved patient outcomes. His program office builds enterprise-wide analytics and improvement capabilities to improve clinical outcomes and foster continuous quality improvement, transparency and organizational learning. Programs under his purview also coordinate patient experience assessment in support of patient- centered care and public reporting of VHA performance data as part of agency efforts to promote informed patient decisions as well as advance government transparency and accountability.

### Sharon Goodman

Ms. Sharon Goodman is a Senior IT Analyst for the Department of Veterans Affairs’ Office of Information & Technology’s Enterprise Command Center division. She worked with the Veterans Healthcare Administration’s Performance Measurement team for over 20 years where she managed clinical data quality, clinical measurement report design, and provided support for the group’s portfolio of clinical performance metrics. Over the years, she also provided services such as business analysis, data management, systems design, technical writing and training. Ms. Goodman holds a degree in Business Management with a specialization in Operations Analysis from Hofstra University in New York.

### Megan Gregory, Ph.D.

Dr. Megan Gregory is an Assistant Professor in the Department of Biomedical Informatics and with the Center for the Advancement of Team Science, Analytics, and Systems Thinking in Health Services and Implementation Science Research (CATALYST) at The Ohio State University College of Medicine. Her research examines individual, social, and organizational factors and conditions that impact health care teams. Dr. Gregory also seeks to develop, implement, and evaluate interventions, tools, and educational programs (including simulation) to improve health care teamwork.

Prior to joining The Ohio State University, Dr. Gregory was an Advanced Fellow in Health Professions Education, Evaluation and Research at the Center for Innovations in Quality, Effectiveness & Safety with the Michael E. DeBakey VA Medical Center and Baylor College of Medicine in Houston, Texas. She received her Ph.D. in Industrial-Organizational Psychology from the University of Central Florida, where she worked for the Institute for Simulation and Training.

### Paul Haidet, MD, MPH

Dr. Paul Haidet is a general internist, health-services researcher, educator, and lover of jazz music. After completing his residency in internal medicine at the Milton S Hershey Medical Center and Penn State University Hospital, he pursued the Harvard Faculty Development Fellowship in General Internal Medicine at the Beth Israel Medical Center in Boston, MA, and completed his Masters in Public Health at the Harvard School of Public Health. He joined the faculty of the Baylor College of Medicine and was a member of the Health Services Research and Development Center of Excellence at the Michael E DeBakey Veterans Affairs Medical Center in Houston Texas. After 11 years at Baylor, Dr. Haidet was relocated back to Penn State, where he inaugurated the role of Director of Medical Education Research and serves as Professor of Medicine, Humanities, and Public Health Sciences at the Penn State College of Medicine. Dr. Haidet has published on a variety of topics related to humanistic care of patients. His current interest is in the improvisational aspects of patient-physician communication. He is using jazz to create new educational methods and models to foster advanced communication skills among physicians.

### Jason R. McKnight, MD, MS

Dr. Jason McKnight is an assistant clinical professor of primary care medicine with the Texas A&M University Health Science Center College of Medicine and the Texas A&M Family Medicine Residency program. His primary responsibilities include both graduate and undergraduate medical education in the fields of hospital medicine, primary care endoscopy, physician advocacy, population health, and preventative care medicine. He is the primary investigator (PI) of a grant from the Cancer Prevention & Research Institute of Texas (CPRIT) entitled “Advancing an established colorectal cancer prevention program for rural and underserved Texans through A&M’s family medicine residency” that has provided and continues to provide hundreds of screening and diagnostic colonoscopy procedures to rural and underserved patients. Dr. McKnight also serves on the Texas Medical Association (TMA) Committee on Rural Health, which seeks to improve the health care resources and infrastructure of rural Texas through policy and legislative changes. He graduated from medical school at the University of Texas-Houston Medical School (McGovern Medical School) with subsequent family medicine residency training at Texas A&M Family Medicine residency program.

### Daniel R. Murphy, MD, MBA

Dr. Daniel Murphy is an Assistant Professor and board-certified Internal Medicine physician at the Baylor College of Medicine. He serves as Medical Director of the General Internal Medicine clinic and Chair of the Quality and Safety Committee. He additionally works with the Patient Safety Center of Inquiry team at the Houston Center for Innovations in Quality, Effectiveness and Safety at the Michael E. DeBakey VA Medical Center. Dr. Murphy has a Master’s in Business Administration from the University of Miami School of Business with a focus on management science and process improvement.

Dr. Murphy’s research interests include understanding workflows related to electronic communication and developing methods to detect/reduce delays in diagnosis and treatment related to miscommunication. His focus has been directed towards measuring and reducing information overload to providers from electronic health records. With his clinical, research and administrative roles, Dr. Murphy seeks to use his research, quality improvement, and clinical practice experience to steer quality and safety efforts.

### Karin Nelson, MD, MSHS

Dr. Karin (“Kari”) Nelson is a general internist and health services researcher at the VA Puget Sound Health Care System. She is a Professor in the School of Medicine, Adjunct Professor in the School of Public Health at the University of Washington and core investigator at the Seattle Health Services Research & Development (HSR&D) Center of Innovation (COIN). Dr. Nelson is the director of the Primary Care Analytics Team for the VHA’s Office of Primary Care. She won the 2015 VHA HSR&D’s Best Research Paper of the Year Award for Nelson, et al. “Implementation of the Patient Centered Medical Home (PCMH) in the Veterans Health Administration (VHA)” published in *JAMA Internal Medicine*. Her primary research interest is in improving disease self-management for disadvantaged populations, focusing on peer support interventions. She actively mentors many junior faculty and is Co-Director for the MD fellowship program in the Seattle HSR&D COIN. Dr. Nelson received her medical degree from the University of Minnesota, and her masters in health services from UCLA.

### Edward Post, MD, Ph.D., FACP

Dr. Edward Post is the National Medical Director for the Primary Care-Mental Health Integration (PC-MHI) program in the Office of Primary Care at VA Central Office, and is field-based at the Ann Arbor VA Healthcare System where he is a practicing primary care physician. He is also Professor of Internal Medicine at the University of Michigan Medical School, where his research interests include mental health services, medical comorbidity, and collaborative disease management in primary care.

# Research Team Roster

### Taylor Hernandez

Ms. Taylor Hernandez is a Research Coordinator in the Department of Medicine at the Center for Innovations in Quality, Effectiveness, and Safety. Taylor balances her time between research and working for the IQuESt operation’s dept. as a Program Service Assistance. She graduated in 2017 with a Bachelor of Science in Management Information Systems. She has her Masters in Healthcare Administration. Her area of interest is patient safety and improving the quality of patient care.

### Ashley M. Hughes, Ph.D.

Dr. Ashley Hughes is an Assistant Professor in the Department of Biomedical and Health Information Sciences at the University of Illinois at Chicago and a Research Health Scientist at the Center for Innovations in Chronic, Complex Healthcare (CINCCH) at the Edward Hines JR VA Medical Center. Holding her doctorate in Human Factors with a master in modeling and simulation from University of Central Florida, Dr. Hughes seeks to better understand and improve the way that healthcare teams leverage health information technologies to coordinate care. Her work has been recognized for its impact and methodological rigor by the Society for Industrial and Organizational Psychology (a field responsible for advancing team science), Human Factors and Ergonomics Society, as well as Indiana University School of Medicine and Duke University’s division of Social Networks and Health.

### Sylvia J. Hysong, Ph.D.

Dr. Sylvia J. Hysong is a Lead Research Scientist at the Center for Innovations in Quality, Effectiveness and Safety (IQuESt, a Center of Innovation sponsored by the U.S. Department of Veterans Affairs Health Services Research and Development Service), where she directs their PhD Post-doctoral fellowship programs, Professor of Medicine at Baylor College of Medicine, and co-director of the VA Quality Scholars Program Coordinating Center. She is an industrial/organizational (I/O) psychologist with two decades of experience in implementation and organizational research. Currently the principal investigator of two federally funded grants, her research interests include primary health care as a work environment, performance measurement, feedback systems, and team coordination. She is the author of 46 peer-reviewed publications, over 120 national and international presentations, and serves as a member of the Department of Veterans Affairs Health Services Research and Development Scientific Merit Review Board. She is also an accomplished educator, and was the recipient of three Norton Rose Fulbright Faculty Excellence Awards for Excellence in Educational Leadership, Teaching and Evaluation, and Educational Research, respectively. Dr. Hysong received her doctorate in 2000 in I/O Psychology from Rice University and completed her Health Services Research post-doctoral fellowship in 2007 at IQuESt.

### Houston F. Lester, Ph.D.

Dr. Houston F. Lester is an Instructor at Baylor College of Medicine and investigator at the Center for Innovations in Quality, Effectiveness, and Safety (IQuESt). He is a quantitative psychologist with a background in industrial/organizational psychology. He completed his Ph.D. in Educational Psychology with a specialization in Quantitative, Qualitative, and Psychometric Methods in 2017 and has a master’s in industrial/organizational psychology from Auburn University. He has 15 peer reviewed publications, and 21 national and local presentations. He is an expert in multilevel, longitudinal, and structural equation modeling and has been asked to give statistical training workshops at universities in the United States and abroad.

### Varsha Modi

Mrs. Varsha Modi is the lead project coordinator for the recently funded AHRQ RO1 Grant entitled Impact of Team Configuration and Team Stability on Primary Care Quality She manages the daily activities supporting this team-centric *Innovative Research in Primary Care* project. The study addresses how different configurations of primary care teams affect the effectiveness and efficiency of care and health outcomes. In addition to her role in the RO1, she supports Dr. Hysong with three other studies involving care coordination. Her area of interest is patient safety and improving the quality of patient care. Ms. Modi has been a staff member at IQuESt for almost 10 years and has been the recent recipient of the FY18 Research Week Service Award for outstanding contributions to Veterans and the Michael E. DeBakey Veterans Affairs Medical Center and recognized for outstanding achievement and exceptional commitment to teamwork.

### Frederick L. Oswald, Ph.D.

Dr. Fred Oswald (Professor, Department of Psychological Sciences) has expertise, 100+ publications, and extensive grant experience in the areas of workforce readiness, personnel selection, college admissions, and measure development (see workforce.rice.edu). Dr. Oswald is active in the National Academy of Sciences (NAS) with regard to these issues, having recently led a workshop on the modernization of personnel selection in the forensic sciences; having participated in two recent NAS reports on measuring human capabilities and on intra- and interpersonal competencies that contribute to success in college students and in the military, respectively; and as a current chair of the NAS Board on Human Systems Integration (BOHSI), which applies systems-level thinking to human interactions in the workplace, with regard to automation, safety, teamwork, and other workplace phenomena. Currently, he is Senior Associate Editor of the Journal of Management; Associate Editor of Psychological Methods; and Associate Editor of Advances in Methods and Practice in Psychological Science; and in 2017-2018, he served as President of the Society for Industrial and Organizational Psychology, a 10,000-member international organization dedicated to the science of workplace phenomena. Dr. Oswald is a fellow of the American Psychological Association (APA, Div. 5, 8, 14), American Psychological Society, and the Society for Industrial and Organizational Psychology.

### Laura A. Petersen, MD, MPH, FACP

Dr. Laura Petersen is a Board-certified internist, a Professor of Medicine and Chief of the Section of Health Services Research at Baylor College of Medicine (BCM).  She is the Director of the Houston Center for Innovations in Quality, Effectiveness, and Safety (IQuESt), one of the nation’s 18 competitively funded VA health services research and development centers of innovation, and Associate Chief of Staff for Research and Development for the Michael E. DeBakey VA Medical Center.  Her research interests focus upon assessing the effects of health care policy and organization on the quality and safety of health care. She is a graduate of the VA HSR&D Career Development Award Program.  Her CV lists 167 peer-reviewed publications, including publications in high impact journals.  Her work has been cited extensively in three different National Academy of Medicine reports, including Rewarding Provider Performance.  She has worked collaboratively with the VA health care system to design and implement research that can improve care in a process that embodies the goals of a learning health care system.  She has four primary BCM faculty mentees, all of whom have held federal career development awards, have independent federal funding as PI, and have been promoted to Associate Professor or Professor.  All her faculty mentees have independent funding as PI, all have been promoted with tenure, and all have begun to mentor junior faculty and fellows who are preparing career development award applications. Dr. Petersen has served in national leadership roles through membership on the National Advisory Committee for the Robert Wood Johnson Foundation Physician Faculty Scholar Award Program, the External Advisory Board for the National Heart, Lung, and Blood Institute (NHLBI) Centers for Cardiovascular Outcomes (CCOR), the American College of Physicians Performance Measurement Committee, and other organizations.  She was the first ever Robert Wood Johnson Foundation Generalist Physician Faculty Scholar at BCM.  She received the American Heart Association Established Investigator Award in 2005, the VA Under Secretary’s Award for Outstanding Achievement in Health Services Research in 2017 and was elected to membership in the Association of American Physicians in 2011.

### LeChauncy Woodard, MD, MPH

Dr. LeChauncy Woodard, is a general internist and Clinical Professor in the Department of Health Systems and Population Health Science at the University of Houston College of Medicine. She also serves as the Founding Director of the Humana Integrated Health Systems Sciences Institute. Dr. Woodard is a clinician educator and health services researcher with extensive experience in interprofessional team-based care, quality improvement, and patient safety. She is also an adjunct faculty member at Baylor College of Medicine and the Center for Innovations in Quality, Effectiveness, and Safety, where she served as principal investigator on several federally funded grants, including the Houston VA Center of Excellence in Primary Education. This interprofessional training program integrated trainees from medicine, mental health, nursing, social work, and pharmacy to provide patient-centered, team-based care to high complexity Veteran patients.

She also served as Associate Director of the VA Quality Scholars Coordinating Center, an interprofessional fellowship program which trains physicians and doctoral-level nurses in quality improvement and patient safety at sites across the United States and in Toronto, Canada. Her research focuses on performance measurement and improving quality of care for chronically ill, multimorbid adults through team-based behavioral health interventions to enhance collaborative goal-setting. Dr. Woodard is committed to advancing the university’s mission to address health disparities and increase the value of health care for patients both locally and nationally.
